# A Review of Radiotherapy for Merkel Cell Carcinoma of the Head and Neck

**DOI:** 10.1155/2012/563829

**Published:** 2012-11-20

**Authors:** Justin Lee, Ian Poon, Judith Balogh, May Tsao, Elizabeth Barnes

**Affiliations:** Odette Cancer Centre, Sunnybrook Health Sciences Centre, Department of Radiation Oncology, University of Toronto, Toronto, ON, Canada

## Abstract

Merkel cell carcinoma of the head and neck (MCCHN) presents a clinical challenge due to its aggressive natural history, unpredictable lymphatic drainage, and high degree of treatment related morbidity. Histological examination of the regional lymph nodes is very important in determining the optimal treatment and is usually achieved by sentinel lymph node biopsy. Radiotherapy plays a critical role in the treatment of most patients with MCCHN. Surgery with adjuvant radiotherapy to the primary tumour site is associated with high local control rates. If lymph nodes are clinically or microscopically positive, adjuvant radiotherapy is indicated to decrease the risk of regional recurrence. The majority of locoregional recurrences occur at the edge or just outside of the radiation field, reflecting both the inherent radiosensitivity of MCC and the importance of relatively large volumes to include “in-transit” dermal lymphatic pathways. When surgical excision of the primary or nodal disease is not feasible, primary radiotherapy alone should be considered as a potentially curative modality and confers good loco-regional control. Concurrent chemoradiotherapy is well tolerated and may further improve outcomes.

## 1. Introduction

Merkel cell carcinoma (MCC) of the skin is an uncommon, neuroendocrine malignancy often associated with a rapidly progressive primary tumour, regional nodal disease, and a high risk of distant metastases. The overall incidence is low, approximately 0.44 cases per 100 000 but appears to be increasing with the aging population [1]. The overall cure rates for MCC are approximately 50% with a high degree of variability amongst reported series based on stage, patient comorbidities, and treatment factors [2]. The most common primary tumour location in 3870 cases reviewed from the Surveillance, Epidemiology, and End Results (SEER) database was the head and neck, which represented half of all cases, among which lesions located on the face were the most common (29%). 

The management of cancer of the head and neck region presents unique challenges to diagnosis and treatment. Given the high predilection of MCC to originate in these regions, the purpose of this study is to evaluate and review the role of radiotherapy in the management of Merkel cell carcinoma of the head and neck (MCCHN). 

## 2. Background

The role of radiotherapy (XRT) in the treatment of nonmelanoma skin cancers of the head and neck is well established. Squamous cell carcinoma (SCC) and basal cell carcinoma (BCC) of the face, scalp, ear, lip, nose, and neck can be treated with radiotherapy alone, with a high cure rate, in excess of 90% in most series [3, 4]. Cosmetic outcomes are generally good or acceptable [5, 6] and the risk of severe, late toxicity such as chronic ulceration, osteoradionecrosis or chondronecrosis are very uncommon and are more likely to be associated with hypofractionated regimens which utilize a high daily radiation dose over a short number of treatments [7, 8]. There are limited data describing the use of radiotherapy for MCC of the head and neck due to the relatively low incidence and lack of prospective clinical trials. Therefore, some overarching principles and lessons learned from decades of treatment of NMSC and SCC of the larynx/pharynx and neck may be cautiously extrapolated to help guide radiotherapy treatments for Merkel cell carcinoma of the head and neck, while recognizing that the radiobiology, patterns of spread, and natural history are unique to this neuroendocrine skin tumour. 

## 3. Staging and Investigations

Clinical examination must include assessment and measurement of the primary tumour as well as palpation of the lymph node regions at risk. In addition, the physical examination must include a check of the surrounding adjacent skin to rule out “in-transit” or satellite metastases which are described in the TNM classification system [9] as “distinct from the primary lesion and located between the primary lesion and the draining regional lymph nodes, or distant to the primary lesion” and denote stage N2 disease.

Patients diagnosed with MCCHN should undergo diagnostic imaging using contrast enhanced CT or MRI to delineate the extent of local disease, assess perineural or bone invasion, and identify in-transit metastases. Regional lymph node involvement may also be assessed by standard imaging but may be insufficient for detecting small volume disease (Sentinel Lymph Node Biopsy is discussed below). Because a high proportion of patients may present with distant metastases, all patients should undergo staging CT scans of the chest and abdomen. Positron emission tomography (PET) has also been shown to be sensitive to MCC. A retrospective review of 18 patients with MCC by Concannon et al. [10] demonstrated that PET detected all tumours as small as 5 mm and that the PET scans altered staging in 33% of patients. 

### 3.1. Sentinel Lymph Node Biopsy (SLNBx)

SLNBx plays a critical role in the management of MCCHN. First, SLNBx has higher sensitivity for detecting microscopic lymph node metastasis. Several single institution reports demonstrate that in patients with MCC who are clinically node negative including CT imaging in some series, the SLNBx was positive in 29–50% of cases [11–14], resulting in upstaging the disease and presumably altering the management of the regional lymph nodes.

Second, the lymphatic drainage patterns of cutaneous tumours of the head and neck region are difficult to predict on the basis of the anatomic location of the primary, with a high degree of variability between patients. Klop et al. [15] compared the actual sentinel lymph node locations with the clinically predicted locations based on lymphatic mapping guidelines for 65 patients with cutaneous melanoma of the head and neck. The authors reported 23% of SLNBx locations to be discordant with the “expected” locations based on classical drainage patterns. In a similar study, Lin et al. [16] found discordance between the “clinically predicted” lymph node region and the actual SLNBx location in 49 of 114 cases (43%) of patients with melanoma of the head and neck region. In these discordant cases, the SLNBx may reveal lymph node drainage patterns that would not typically be included in standard neck dissection or radiation field, for example, postauricular nodes, inferior or posterior neck, and even contralateral nodal basins. Despite these advantages, it is also important to consider limitations of SLNBx in the head and neck region. The false negative rates in the head and neck region are known to be higher than in other anatomic sites, due to multiple pathways or aberrant drainage basins. SLNBx in a postoperative setting may also be affected by disruption of the tissues and lymphatics in the primary tumour bed. An extensive systematic review of 3442 patients included in 32 studies demonstrated a median false negative rate for nodal recurrence of 20.4% [17]. Although there are limited data examining the predicted versus actual lymphatic drainage patterns in MCCHN, it would seem reasonable to expect that these tumours may also exhibit a relatively high degree of variability.

Overall, despite those limitations described, SLNBx has the ability to improve nodal staging accuracy and guide radiotherapy or surgical treatment decisions in patients with MCCHN and is recommended for the vast majority of patients. One exception may be patients with very small tumours <1.0 cm, who are reported to be at a very low risk (4%) of regional nodal metastasis [18]. 

## 4. Radiotherapy for Merkel Cell Carcinoma of the Head and Neck

Merkel cell tumours are known to be highly radiosensitive and often exhibit dramatic response to moderate doses of XRT. Radiotherapy treatment of both the primary tumour site and regional lymphatics should be considered and discussed for all patients, ideally in a multidisciplinary case conference. The most common role of XRT is in the adjuvant setting but in cases where surgery is not feasible or declined, primary radiotherapy represents an alternative option for curative treatment. XRT is typically administered in a standard, conventional fractionation schedule, 1.8–2 Gy daily over 5 to 7 weeks, as normal tissue tolerances of the head and neck are well established and there is no evidence to suggest a benefit for altered fractionation. Due to the low incidence of MCC, most reports on the subject group together all anatomic locations for analysis. This more general data is presented below and when available, specific evidence pertaining to MCC of the head and neck is highlighted. 

### 4.1. Adjuvant Radiotherapy to the Primary Site

To our knowledge, there are no prospective randomized studies examining the use of postoperative adjuvant radiotherapy in the setting of MCC. A large number of reports have been published on this topic, including patients who underwent wide local excision (2–5 cm margins) with and without adjuvant radiotherapy with mixed results. A relatively large, single institution study of MCC [19] including all locations found a low rate of local recurrence (8%) when negative margins were achieved and the nodal recurrence rate was as low as 11% in patients who had undergone SLNBx or nodal dissection. Seventeen percent (41 of 237) of patients underwent adjuvant radiotherapy and the authors did not detect a significant difference in locoregional recurrence rates associated with XRT. It should be noted that the mean diameter of tumours located in the head and neck in the study was 1.3 cm and only 16% of MCCHN patients presented with clinically involved lymph nodes. In contrast, a multicentre, retrospective study of 110 patients with MCC [20] specifically located in the head and neck region found that combined modality treatment with surgery and radiotherapy was associated with better local and regional control, compared with either surgery or radiation alone. Lawenda et al. [21] also found a statistically significant improvement in local control rates associated with adjuvant XRT to the primary site compared with surgery alone (95% versus 69%, *P* = 0.020) in a series of 36 patients who all had MCCHN. 

A meta-analysis of 132 studies yielded 1254 eligible patients with a single primary Merkel cell carcinoma lesion of the skin treated with surgery [22]. Approximately two thirds of patients underwent surgery alone, (the rest had surgery and adjuvant radiotherapy) and all patients included in the meta-analysis had negative margins. Patients treated with adjuvant radiotherapy had a statistically significant reduction in both local (hazard ratio, HR = 0.27, *P* < 0.001) and regional recurrence rates (HR = 0.34, *P* < 0.001). Cause specific and overall survival reached statistical significance favouring adjuvant XRT only when single case reports and studies involving a single-treatment group were excluded.

Another large review of patients with MCC was reported by Mojica et al. [23] based on the Surveillance, Epidemiological, and End Results (SEER) database. 1487 patients with MCC stage I–III, were included in the analysis and 40% of those patients had adjuvant XRT. Patients who received adjuvant radiotherapy had a statistically significant difference in overall median survival compared with those who had surgery alone (63 versus 45 months, *P* = 0.0002) despite the fact that the irradiated group had a higher incidence of regional disease at diagnosis. Subset analysis suggested that patients with tumour size >2 cm derived the greatest benefit from adjuvant XRT. 

The current evidence suggests that adjuvant radiotherapy to the primary tumour bed should be recommended in most patients with MCCHN following surgery, to decrease the risk of local recurrence. It is possible that patients with small, pathologically node-negative tumours that have been widely excised with negative margins >3 cm may be at sufficiently low risk to consider observation but identification of these very low risk patients with tumours of the head and neck region is likely to be imprecise and infrequent. 

### 4.2. Adjuvant Radiotherapy of Regional Lymph Nodes

MCCHN should be assessed with CT and/or MRI of the regional lymph nodes as well as sentinel lymph node biopsy. The importance of SLNBx in MCC is described above and also supported by a review of the SEER database which identified 2104 patients who had specifically MCC of the head and neck [24]. The *absence of histologic lymph node evaluation* was an independent prognostic factor for disease specific survival, suggesting that patients may have benefited from additional nodal treatments. Patients with lymph node positive disease will typically proceed to have completion neck dissection. 

The role of adjuvant radiotherapy to the regional lymphatics after SLNBx or complete nodal dissection is not well defined. Veness found a high risk of regional lymph node recurrence (43%) in patients with clinically positive lymph nodes after surgical dissection alone, compared with 14% risk recurrence rate in patients undergoing dissection followed by adjuvant radiotherapy to the nodal basin [25]; in addition the authors found in a separate series of 37 patients who had exclusively head and neck MCC [26] that nodal radiotherapy was associated with a lower risk of regional relapse. Allen et al. [19] also observed a high rate of regional recurrence rate of 26% in patients with clinically node positive disease treated with surgical dissection alone. Lok and colleagues [27] also focused on the use of radiotherapy for patients with Merkel cell carcinoma of the head and neck. Only 6% of patients (3 out of 48) developed locoregional failure following surgery and radiotherapy for MCCHN with a median followup period of 51 months. All 3 regional recurrences occurred outside of the radiation field, reinforcing the importance of accurate target delineation of regions at risk. 

In the only prospective randomized study of radiotherapy in MCC reported to date, investigators evaluated the efficacy of “adjuvant prophylactic regional radiotherapy” in patients with Stage 1 Merkel cell carcinoma [28]. Although the trial was not completed as planned due to increasing use of sentinel lymph node biopsy, results from 83 patients were evaluated and demonstrated a significant reduction in regional recurrence (16.7% versus 0%, *P* = 0.007) favouring regional irradiation. Based on the available evidence it is useful to group the management of regional lymphatics in MCCHN into commonly encountered clinical scenarios.

- No histologic examination of regional LNs, no SLNBx, or dissection: if SLNBx is not technically feasible, or the patient declines then the regional lymph nodes should be irradiated. A prospective, randomized study demonstrates significant risk of nodal recurrence (16.7%) even in stage I disease and other retrospective reviews suggested a similar risk of nodal recurrence (17.6%) [13, 28].
- Node negative disease after SLNBx or nodal dissection: although there is a risk of false negative results with SLNBx of the head and neck region, most patients with negative biopsy do not require full regional irradiation of the draining lymphatic basin. A reasonable approach may be to include adjacent lymph nodes that are located within the usual 3–5 cm margin from the postoperative bed. For example, adjuvant XRT for a tumour located in the pre-auricular region may include the superficial lobe of the parotid and upper jugulodigastric nodes but not extend to submandibular region or inferior neck.
- Clinically positive or histologically positive: based on a high risk of regional recurrence with surgery alone and moderate radiation toxicity with intermediate doses of XRT, patients with node positive MCCHN should be recommended to undergo regional irradiation of a minimum of one echelon distal and proximal to the involved node as well as in-transit skin between the primary disease and lymph nodes if clinically feasible [25, 29].

### 4.3. Primary Radiotherapy for Merkel Cell Carcinoma

In cases where MCCHN is not treatable by primary surgery for reasons such as unacceptable deformity or defect, patient comorbidities resulting in unacceptably high perioperative risks or patient refusal of surgery, radiation alone or in combination with chemotherapy may be considered as the primary curative treatment modality. 

Veness et al. reported on the Australian experience of radiotherapy alone for Merkel cell carcinoma [30]; 43 patients underwent radiotherapy as the primary treatment for either new diagnosis or gross tumour recurrence. The median age of patients was 79, and approximately half of all patients had a primary lesion of the head and neck. The in-field control rate was 75% and most recurrences occurred at distant metastatic sites. Pape et al. [31] also observed high locoregional in-field control rates in 25 patients with MCC treated exclusively with radiotherapy. After median followup of 3 years, the XRT-treated patients had no local recurrence and only 2 regional nodal relapses. These results were similar to a matched cohort of 25 patients at the same institution who underwent both surgery and radiation.

A prospective phase II study involving radiotherapy for MCC, by Poulsen et al., studied the use of concurrent chemoradiotherapy consisting of 50 Gy XRT plus carboplatin and etoposide in patients with high risk features [32]. A total of 53 patients were enrolled (22 of whom had MCCHN), 38 were treated postoperatively, and 15 patients had macroscopic disease treated with chemo-XRT without surgery. For the patients undergoing chemo-XRT alone as primary treatment, the 3 year locoregional control rate was 71% and overall survival was 45%. The prospect of radiotherapy mono-therapy has also been examined in the setting of clinically positive lymph nodes. Fang et al. examined results of patients with macroscopic lymph node disease who had either radiotherapy alone or surgery plus nodal irradiation [33]. The rate of locoregional control with XRT alone for clinically positive nodes was 78% at 2 years, compared with 73% in the cohort receiving surgery plus radiation (*P* = 0.8). 

### 4.4. Radiation Planning: Clinical Target Volume

Ideally, patients with MCCHN should undergo a multidisciplinary assessment prior to surgical excision of the primary lesion. This allows for planning of the sentinel lymph node biopsy, surgical planning for the best cosmetic outcome and also radiation oncology assessment and documentation of the primary tumour location and features. Preoperative clinical photographs are also often helpful in determining the adjuvant radiotherapy field. The radiation target volume must be carefully considered on an individual case by case basis. General principles of target coverage apply to the primary site, lymphatics and perineural involvement.

- Adjuvant XRT for Primary Tumour Site: Target includes the scar, postoperative bed and an additional margin of 3–5 cm where clinically feasible. Adjuvant XRT to the primary site will often include the immediately adjacent nodal regions.
- Regional Lymph Node XRT: at a minimum, the levels of the head and neck directly adjacent to the involved nodes (i.e., one level proximal and distal to involved regions) should be covered as well as all “in-transit” skin and dermal lymphatics between the primary tumour and draining nodal basin.
- Perineural Invasion: if MCCHN involves named nerves or presents with clinical neurologic symptoms, the radiotherapy target should include the nerve pathways and associated ganglion retrograde to the base of skull foramen as described by others in the management of other cutaneous malignancies of the head and neck [34, 35].

### 4.5. Radiation Dose

The optimal radiation dose for treatment of MCCHN has not been studied in a prospective fashion. One retrospective review of 112 patients with MCC addressed the issue of dose-response in subclinical and gross disease. The authors concluded that doses of ≥50 Gy could be used for microscopic disease and that gross disease should be treated to ≥55 Gy, citing a decreased risk of in-field recurrence with increasing dose [29]. These authors and others have reported local recurrences just beyond the field edge, suggesting the importance of extending primary margins (4-5 cm) rather than dose escalation for microscopic residual [27, 29].

Radiation doses of up to 60–70 Gy are routinely used in the treatment of SCC of the head and neck. Based on available data, we have adopted generally accepted XRT doses for MCCHN.

- Microscopic, subclinical, and high risk regions: 50–56 Gy.
- Primary radiation treatment of locally advanced, gross disease: 60–66 Gy.

### 4.6. Radiation Treatment Toxicity

Radiation skin toxicity is related to the total dose, volume, surface area, fractionation schedule, and patient factors such as age, vascular disease, and tumour location. Cumulative experience from the treatment of nonmelanoma skin cancer indicates virtually all patients will develop at least mild-to-moderate acute side effects such as erythema, pruritus, desquamation [36] as well as possible late radiation changes such as atrophy, change in pigmentation, telangiectasia, or fibrosis. Serious or severe complications associated with skin radiotherapy such as chronic ulceration, necrosis of the skin, bone, or cartilage requiring surgical repair are rare (approximately 1%), and may be associated with reirradiation or hypofractionated treatment schedules [7, 8, 37].

## 5. Conclusions

Radiotherapy plays a critical role in the management of Merkel cell carcinoma of the head and neck. Local adjuvant treatment to the primary tumour site and any positive lymph node regions is associated with lower rates of locoregional recurrence. Sentinel lymph node biopsy should be considered standard of care for these patients due to the high risk of occult metastases and difficulty in accurately predicting lymph node drainage patterns. The patterns of recurrence in this challenging disease reinforce the importance of wide radiation margins and consideration of in-transit or satellite metastases. For patients with inoperable or unresectable disease, the available evidence suggests that radiotherapy as the primary therapeutic modality results in tumour control rates which are comparable to surgical series. 
